# Exploring the microbiome of two uterine sites in cows

**DOI:** 10.1038/s41598-023-46093-0

**Published:** 2023-10-31

**Authors:** Nilton Luis Murga Valderrama, Gleni Tatiana Segura Portocarrero, Ana Cecilia Romani Vasquez, Hugo Frias Torres, Gary Jacsel Flores Durand, Victor Guillermo Cornejo Villanueva, Jakson Ch. Del Solar, Richard Costa Polveiro, Dielson da Silva Vieira, William Bardales Escalante, Segundo José Zamora-Huamán, Carla Maria Ordinola-Ramirez, Jorge Luis Maicelo Quintana, Rainer Marco Lopez Lapa

**Affiliations:** 1 Instituto de Investigación en Ganadería y Biotecnología, Facultad de Ingeniería Zootecnista, Agronegocios y Biotecnología, Universidad Nacional Toribio Rodríguez de Mendoza de Amazonas, Chachapoyas, 01001 Chachapoyas Peru; 2 Facultad de Ingeniería Zootecnista, Agronegocios y Biotecnología, Universidad Nacional Toribio Rodríguez de Mendoza de Amazonas, Chachapoyas, 01001 Chachapoyas Peru; 3 Laboratorio de Fisiología Molecular, Instituto de Investigación en Ganadería y Biotecnología, Facultad de Ingeniería Zootecnista, Agronegocios y Biotecnología, Universidad Nacional Toribio Rodríguez de Mendoza de Amazonas, Chachapoyas, 01001 Chachapoyas Peru; 4 Laboratory of Bacterial Diseases, Sector of Preventive Veterinary Medicine and Public Health, Department of Veterinary, Universidade Federal de Viçosa, Viçosa, MG Brazil; 5 Basic Medical Sciences, College of Veterinary Medicine, Purdue University, West Lafayette, IN 47907 USA; 6 Chemistry Department, Institute for Drug Discovery, Purdue University, West Lafayette, IN 47907 USA; 7 Facultad de Ciencias de la Salud, Universidad Nacional Toribio Rodríguez de Mendoza de Amazonas, Chachapoyas, 01001 Chachapoyas Peru; 8 Facultad de Medicina, Universidad Nacional Toribio Rodríguez de Mendoza de Amazonas, Chachapoyas, 01001 Chachapoyas Peru

**Keywords:** Microbiology, Systems biology, Zoology

## Abstract

Bacterial communities in the mammalian reproductive system can be rich and diverse, differing in structure and quantity depending on location. In addition, its microbiome is associated with the state of health of this tract and reproductive success. This study evaluated the microbiome composition of the uterine body (UB) and uterine horn mucosa (UH) samples using 16S rRNA sequencing of samples extracted from cows in the Amazon region. It was observed that four main phyla were shared between the uterine sites: *Actinobacteria*, *Bacteroidetes*, *Firmicutes*, and *Proteobacteria*. Linear discriminant analysis effect size and heat tree analysis showed that members of *Lachnospiraceae* (NK3A20 group) and *Oscillospiraceae* were significantly more abundant in the UB than in UH. In addition, there are more unique genera in the UB than in the UH. A higher bacterial load in UB than in UH is expected because of the exposure to external factors of UB. However, comparing the site's communities through beta diversity did not generate well-defined clustering. Thus, it can be attributed to the closeness of the sites, which would make the niches similar ecologically and microbiologically. Therefore, this research provides knowledge to understand biomarkers in the prior reproduction period.

## Introduction

Cattle production contributes to economic development in many countries, particularly in developing nations, where livestock represents a significant share of the agricultural sector^1^. In the Amazon of Peru, crossbreeding between creole breed and specialized bovine breeds (Holstein, Brown Swiss, Angus, Simmental) is widely spread. Creole breed has adaptive advantages such as lower nutrient requirements in their diet and greater longevity under adverse environmental conditions^2^. Hence, crossbreeds can present the adaptive features and productivity features of specialized bovine breeds. In America, breeding techniques have been contributing to the efficiency of reproduction^3^. Although factors such as microbiome are essential to be studied to approach the efficiency of reproduction.

Nowadays, the microbiome inhabits a wide range of niches, including eukaryotic hosts such as cattle^4^. The reproductive tract was once thought to be sterile (without microorganisms). However, studies reported the presence of bacteria in reproductive tissues of the different parts that contain the cow uterus^5,6^. Cow uterus contains the uterine body (UB) and two uterine horns (UHs) which have their own oviduct^7^.

The type and quantity of bacterial taxa vary depending on the location and stage^8,9^. Moreover, microbiome influence on cattle growth is related to several functions such as helping in the digestion and modulation of the immune system^4^.

It is known that the microflora in the reproductive system is a network of interconnected communities of constant exchange^10^. The bacterial communities in the reproductive tract are essential for maintaining pH, nutrient balance, and immune responses, but imbalances can impact host health and fertility. These community dynamics vary throughout the estrous cycle. Understanding these dynamics is crucial for identifying prevention and treatment strategies for reproductive tract-related health issues^11^.

Furthermore, external microorganisms can enter into the female reproductive tract and generate changes in the uterus microbiome. Moreover, it is plausible that uterine pathogens might assist each other in avoiding uterine defense mechanisms and interact to facilitate colonization of the endometrium. Therefore, collectively the co-occurrence of uterine pathogens could be considered of major importance in the development of uterine infection^12^.

In Peru, the majority of cattle studies are based on phenotypic features^13,14^, and some of them include genetic analysis of the cattle^2,15^. Furthermore, the microbiome of organs that are affected by it such as the gut or uterus has been little studied. Additionally, studies of the ruminant uterus microbiome in Peru commonly used microbiological techniques to characterize some bacteria inside the uterus microbiome^16,17^.

The microbial profile in the reproductive system of cows in Peru and the signaling and mechanism behind synergisms need to be elucidated. As said before, a significant imbalance in the microbiota may affect embryo implantation, healthy aspects and commercial aspects of animal production. Therefore, in the present study, we evaluated and compared the microbiome of two segments of the uterus (UB and cranial half of the UH) of cows using 16S ribosomal RNA sequencing.

## Results

### Summary of collecting segments and sequencing

**Figure 1:**
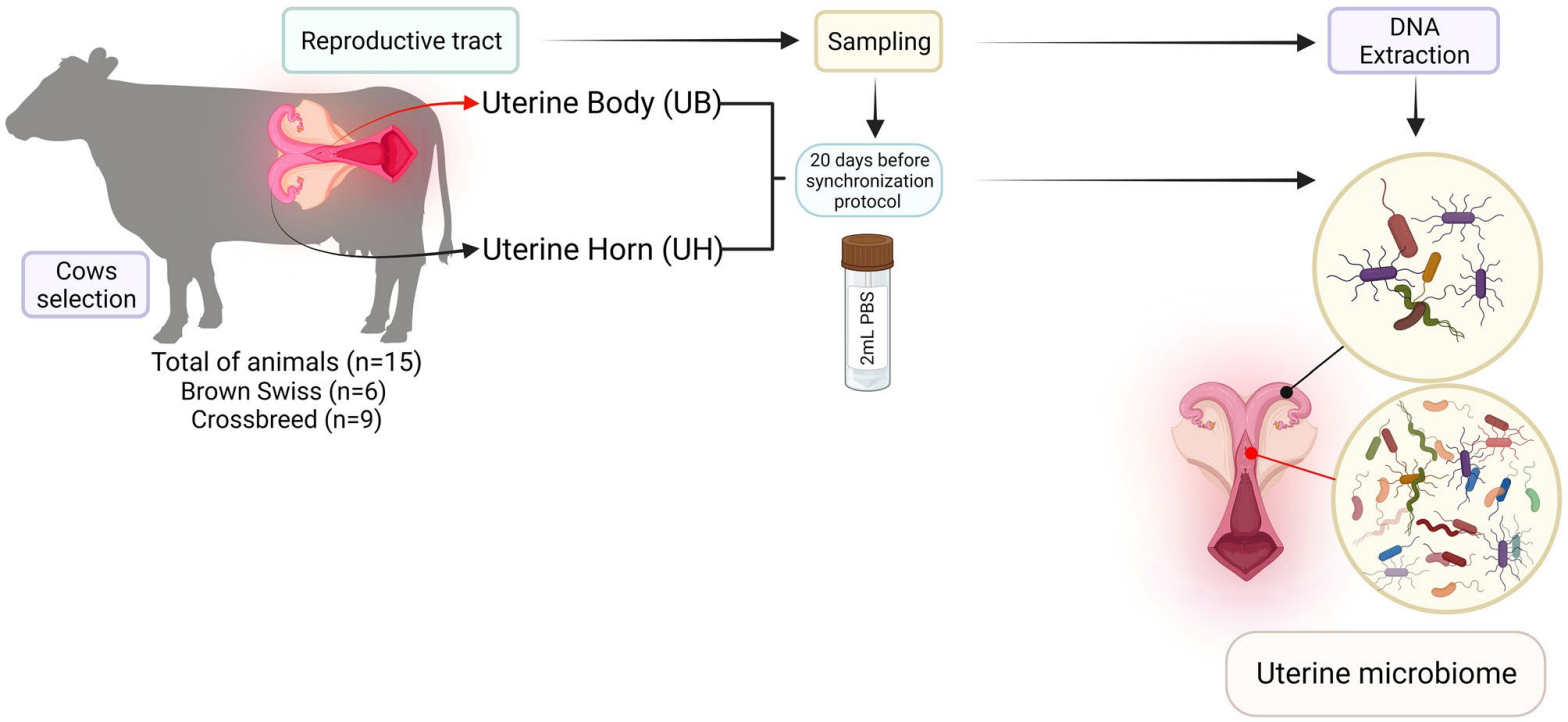
Schematic illustration of the sample extraction. Samples from the Uterine body and Uterine horn were extracted by cytobrushes. The samples were treated with different depletion and extraction methods to obtain the nucleic acids to identify the microbiota of each site.

We used 15 samples: seven samples from UH, and eight samples from UB (Fig. 1). The V4 region was amplified and sequenced in all 15 samples, then quality-filtered reads were demultiplexed, and a total of 1,425,855 sequences were used for downstream analyses [95,057.000 ± 31,078.536 (mean ± SD) reads/sample]. The median length for all reads was 257.97 bp. Overall, 296 taxa identified were used in the analyses.

### Alpha diversity of microbiota in segments of the uterus

**Figure 2:**
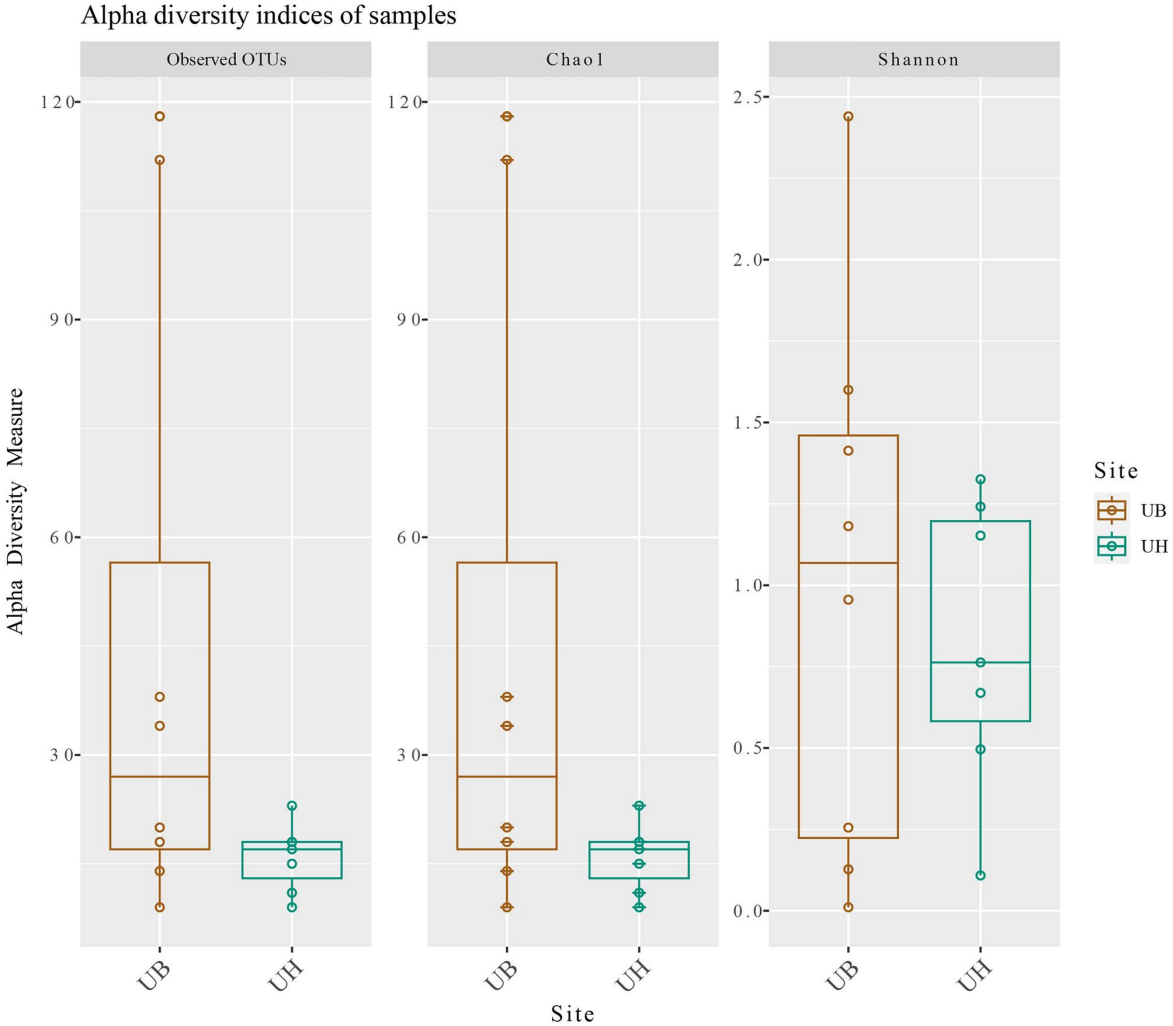
Box plots of alpha diversity indices (Observed OTUs, Chao1 and Shannon) for Uterine Body group (UB) and Uterine Horn group (UH). Different colors indicate different reproductive tract sites (UB, brown; UH, green). The horizontal line inside the boxes represents the median, the thick white box indicates the interquartile range and the thin vertical line represents the rest of the distribution. The complete data analysis of this figure has been stored in the Supplementary File 1.

The Fig. 2 analysis of alpha diversity through the Chao1 and Shannon indices showed that the UB had more richness and diversity than the UH, however this difference did not have significance between the site groups. In addition, the UB had more variance than the UH. In the rarefaction curve, the UH group exhibited a low number of species compared to the UB group and both curves cover all the diversity (Supplementary File 1, Fig. S1).

### Differences in microbial composition among site groups based on beta diversity

**Figure 3:**
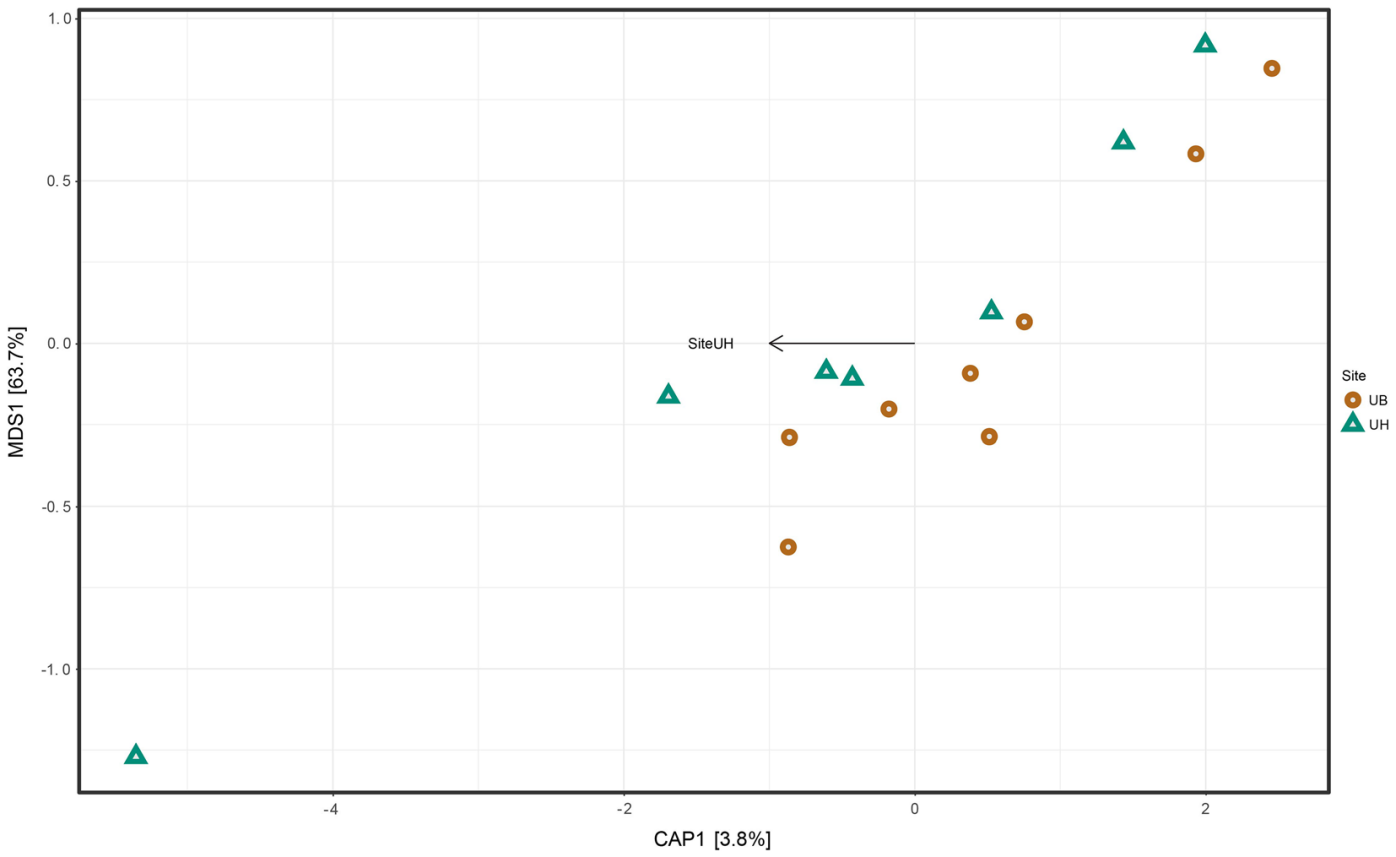
Canonical Principal Coordinate Analysis (CAP) built on an unweighted UniFrac distance with groups of reproductive tract sites: Uterine Body group (UB; circle open circle, green) and Uterine Horn group (UH; triangle open triangle, brown). The forms depict the uterus sites. The order of the arrows demonstrates the formation of groups of individuals selected in different coordinates, denoting the dissimilarity and similarity of microbiota composition among samples and groups, according to the sites.

Analyzing the Beta diversity, we observed that the CAP plot did not give clusters that separate UB and UH (Fig. 3). In addition, the direction of the graphic did not show a visual separation between the UB and UH samples. Likewise, the NMDS plots based on weighted and unweighted Unifrac distances (Supplementary File 1, Fig. S2, S3) did not show clear clustering of the site groups. Besides, the unweighted Unifrac distances did not have a significant difference (*p* = 0.919), also the same happens with weighted Unifrac distances (*R2* = 0.04051; *p* = 0.666). Furthermore, the analysis of similarities showed that both sites have different communities. However, this result was not significant (Anosim of Unifrac unweighted: *R* = −0.06924; *p* = 0.80619, Anosim of Unifrac weighted: *R* = −0.04738; *p* = 0.73726).

### Differences in the composition of the bacterial microbiota for each group

**Figure 4:**
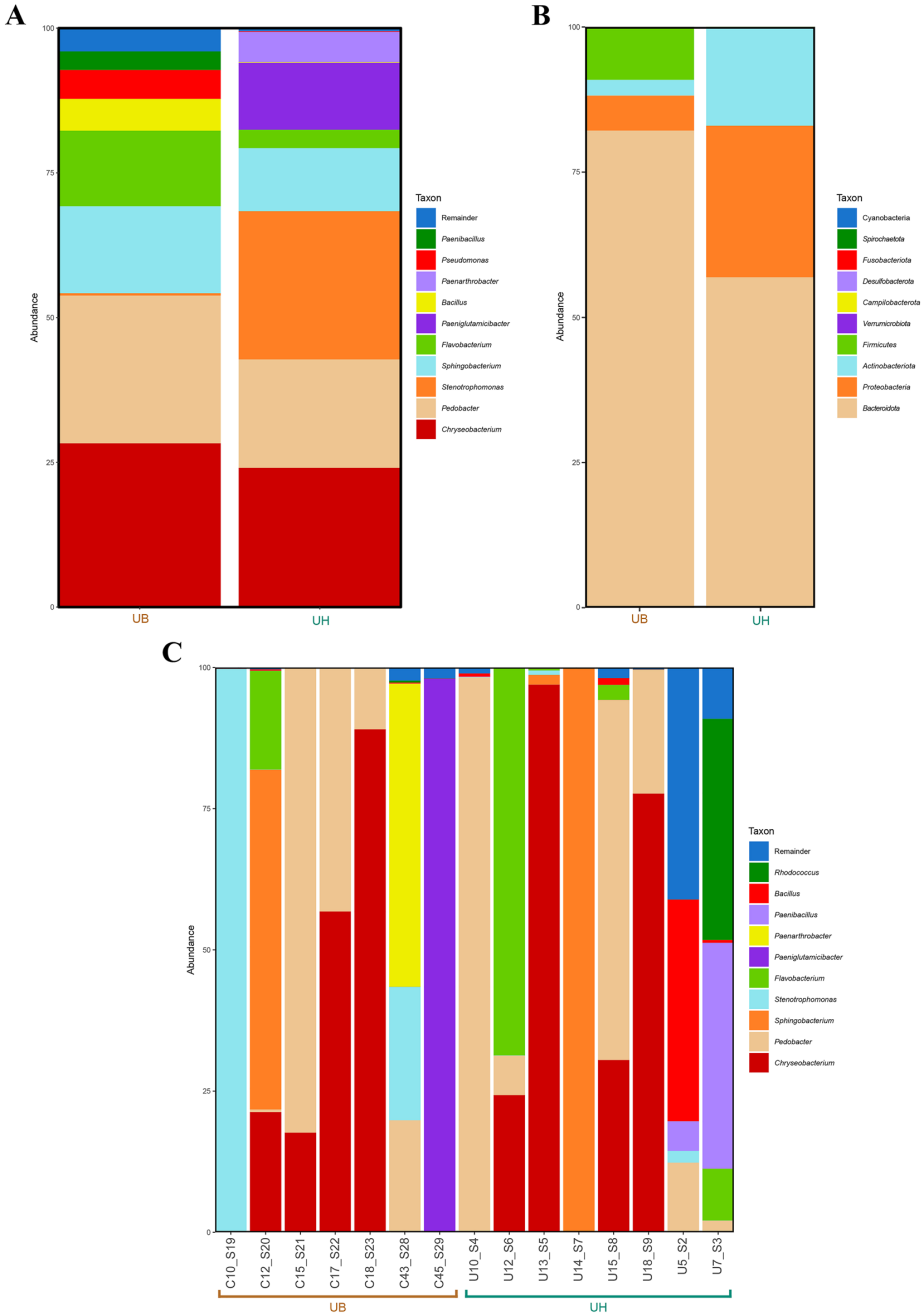
Bacterial microbiota composition in terms of relative abundance at phylum and genus levels, in the Uterine Horn group (UH) and Uterine Body group (UB): **(a)** Taxonomic composition of the ten main bacterial genera with different abundances between the uterus sites, with each color corresponding to a different genus. **(b)** Taxonomic composition of the ten main phyla and differentially abundant bacterial taxa, with each color corresponding to a phylum, in the different sites. **(c)** Taxonomic composition of the ten main genera and differentially abundant bacterial taxa, with each color corresponding to a different genus and subdivided by independent samples. The letter C in the name of the samples stands for the samples from the UH group, and the letter U for the samples of the UB group. The complete data analysis of this figure has been stored in the Supplementary File 2.

We compared the microbial compositions found in animals of site groups based on their relative abundances (Fig. 4). At the genus level, we found that *Chryseobacterium* has the highest abundance among groups, with variable rates of 26.40% and 28.69% for UH and UB, respectively. The most abundant genera after *Chryseobacterium*, for the UB, were *Pedobacter* (25.65%), *Sphingobacterium* (12.70%), *Flavobacterium* (10.12%), *Paenibacillus* (5.66%), *Bacillus* (5.21%) and *Rhodococcus* (4.90%); for the UH, were *Pedobacter* (22.37%), *Stenotrophomonas* (17.59%), *Paeniglutamicibacter* (14.00%), *Sphingobacterium* (8.60%), *Paenarthrobacter* (7.67%) and *Flavobacterium* (2.51%). In addition, we found some genera present in low abundance in only one of the groups, e.g., the genus *Rhodococcus* had an abundance of 0,05% in the UH, but a relative abundance of 4.90% in the UB. The other percentages of the remaining genera and his complete taxonomy are in the Supplementary File 2. At the phylum level, we found that the most abundant phylum in both sites was *Bacteroidotes* (59.91 and 77.36%). However, the following order of the most abundant Phyla variates; in the UB was *Firmicutes* (11.35%), *Proteobacteria* (5.89%), and *Actinobacteria* (5.38%); while in UH was *Actinobacteriota* (21.72%), *Proteobacteria* (18.24%) and *Firmicutes* (0.12%). The absolute microbiota composition figure is displayed in the Supplementary File 1, Fig. S4.

**Figure 5:**
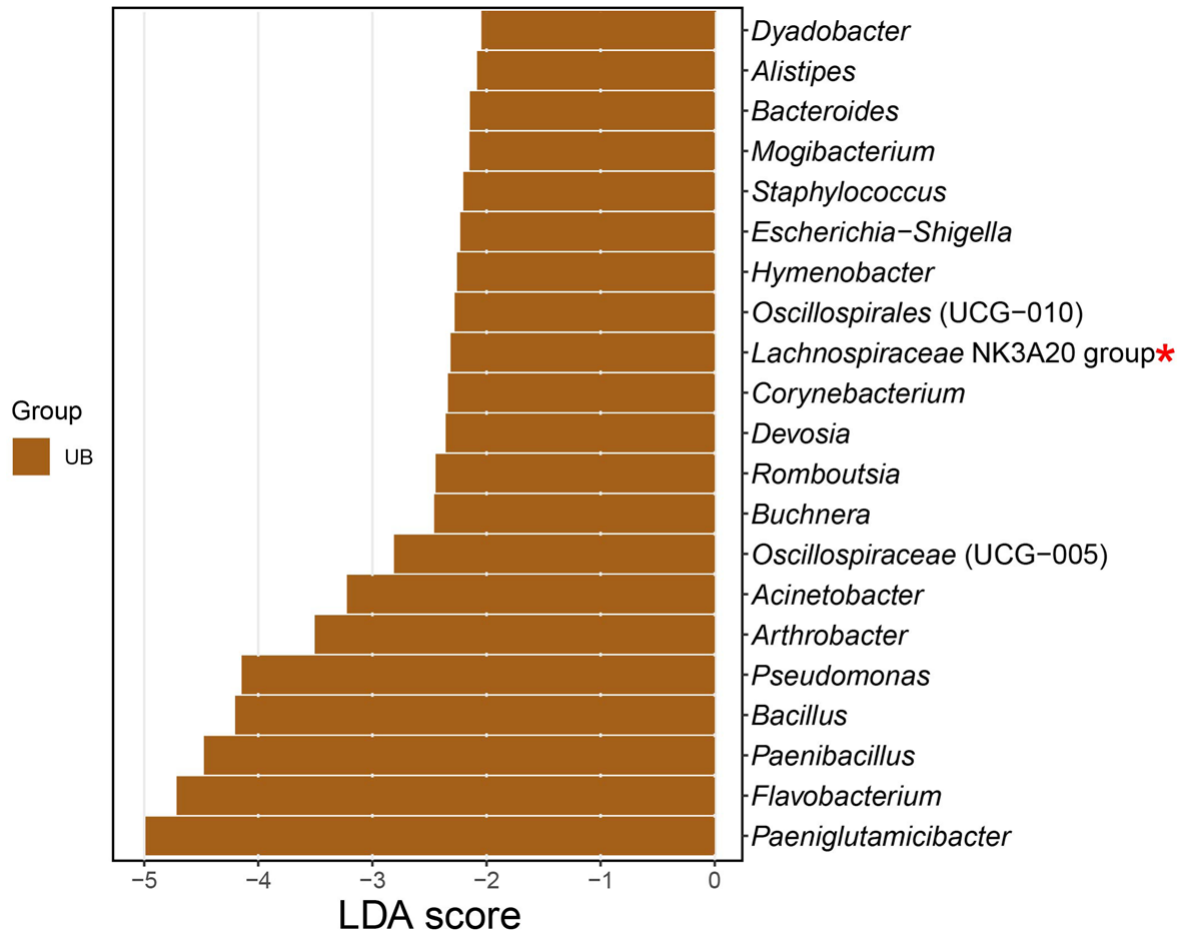
Linear discriminant analysis (LDA) effect size (LEfSe) comparison of differentially abundant bacterial taxa between different groups (UB and UH). Horizontal bars represent the effect size for each taxon: brown color indicates taxa enriched in the Uterine Body group (UB). LDA score cutoff of 2.0 was used to discriminate bacterial taxon. The red asterisk denotes a taxon with a significant difference between the abundances of breed groups (adjusted p-value < 0.05 and unadjusted p-value < 0.05). The complete data analysis of this figure has been stored in the Supplementary File 3.

Linear discriminant analysis (LDA) Effect Size (LEfSe) was performed to identify specific genera that varied in relative abundance consistently in the reproductive tract site (UB and UH). The threshold of LDA score (log10) was 2 (Fig. 5). Enrichment of a total of 21 potential biomarkers (LDA score > 2, LDA score < −2) was found in the UB group. The three bacteria genera with the highest LDA score were *Paeniglutamicibacter*, *Flavobacterium* and *Paenibacillus*.

In addition, only one genus showed a significant enrichment, and it has biologically consistent differences (p-value < 0.05; LDA score > 2, LDA score < −2) in the microbiome of the UB group: *Lachnospiraceae* (NK3A20 group). The complete taxonomy of each genus was detailed in the Supplementary File 3.

### Difference between common taxonomic ranges and identification of unique taxa in site groups

**Figure 6:**
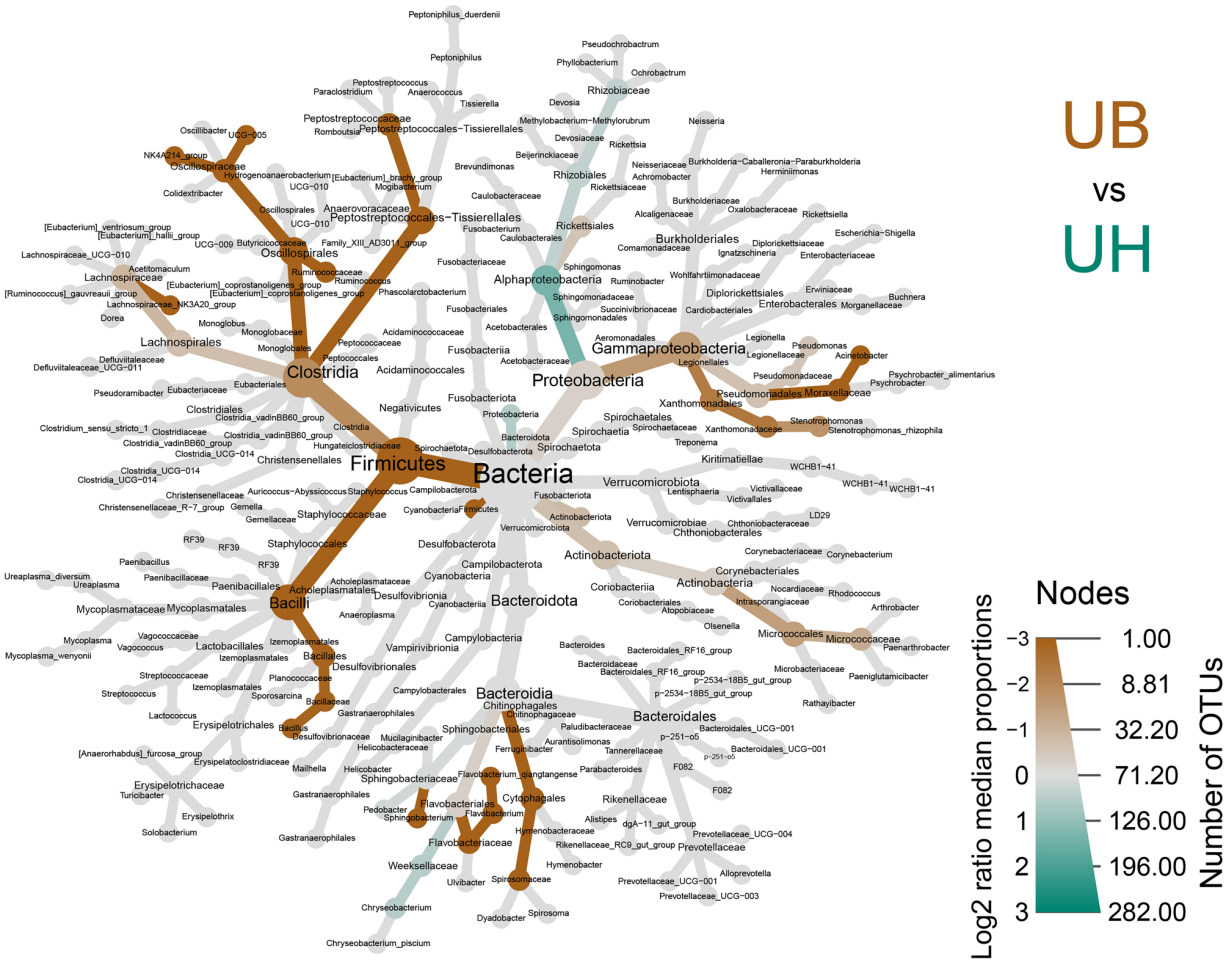
Heat tree illustrating the general taxonomy of the cow mucosa bacterial community in all sites. The heat tree illustrates comparisons between Uterine Body group (UB) and Uterine Horn group (UH). The color intensity is related to the log2-ratio of the difference in median proportions and to the Wilcoxon test applied to the readings among each group. The brown taxa indicate an enrichment of the UB group, and green refers to the UH group. In gray, the nodes are equally present in both compartments. The complete data analysis of this figure has been stored in the Supplementary File 4.

We made a heat tree analysis to compare the abundance of common taxonomic ranges of the site groups (Fig. 6, Supplementary File 4). Among the taxa, we only found two genera that have significant differences between site groups: *Lachnospiraceae* (NK3A20 group) and *Oscillospiraceae* (NK4A214 group).

**Figure 7:**
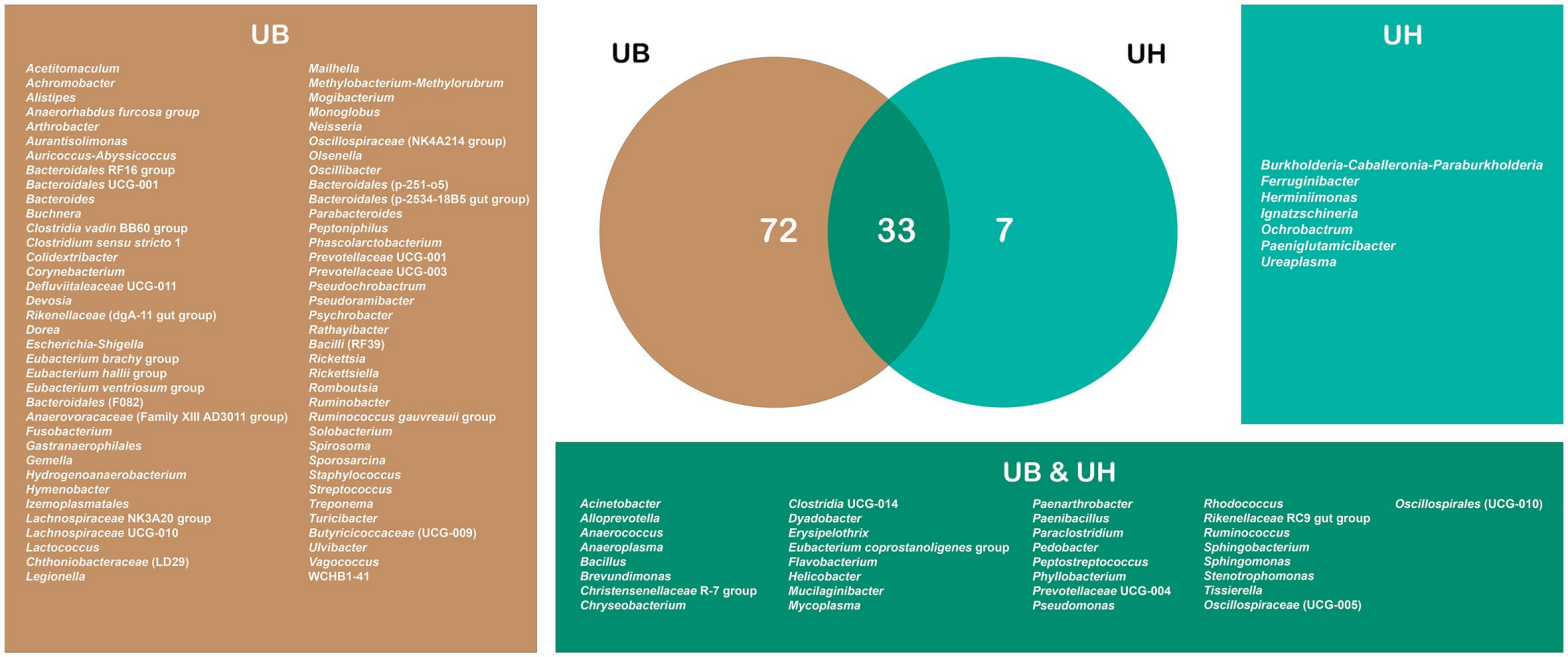
Venn chart with taxa of bacteria divided by site groups. The groups are distributed in different colors: brown for the Uterine Body group (UB); green for the Uterine Horn group (UH). The panel frames are labeled with the colors of their respective groupings of the venn analysis and identify each genus that has been cataloged for their groups. Some genera may have the same name, but they are different amplicon sequence variants (ASVs), that is, different species and subspecies are possible. The complete taxonomy of these taxa was specified in the Supplementary File 5.

The visualization of common and exclusive taxa between uterus sites was shown through a Venn Diagram (Fig. 7, Supplementary File 5). UB presented 72 unique genera and UH presented 7. The number of common genera between both sites was 33. Therefore, the total of exclusive taxa was 79. This result indicates that the majority of taxa in UB were exclusive, while the majority of taxa in UH were common with UB.

## Discussion

The reproductive tract composed of the vagina, cervix and uterus showed differences in their microbiome. The analysis of the richness and diversity showed that the UB has more taxonomic ranges. This result is expected because the UB has more exposure to external factors than uterine horns providing more bacterial load^18^. A study of different sections of vagina and uterus of 110 women demonstrates different bacterial communities according to the sites^19^. In cattle, the environmental exposure of the uterus during calving is associated with the acquisition of environment bacteria, since that exposure generates change after calving into the microbiome and affects the follicular development^20^. Several studies present an association between the microbiome and uterus diseases that affects reproductive success, but these pathogens are not consolidated as the cause of the disease^21,22^. For that reason, the study of the microbiome into different sections of the uterus can help to determinate the features of the microbial community and help to determine potential pathogens that can generate uterine diseases.

In the analysis of beta diversity, clusters are not grouped by site. The weighted and unweighted Unifrac distances between the site groups did not present defined clusters when they generated a PCoA based on distances, and these clusters were so close to each other to differentiate^19^. Hence, the diversity of the samples does not depend on uterine sites. This suggests that the sites are closely related ecologically and microbiologically, indicating similarity in niches. A previous metagenomic study of different sites of the female reproductive tract in humans showed similar results about uterine parts (fallopian tubes, endometrium, pouch of Douglas)^19^.

The most abundant phyla found in this research are similar to several studies. We also identified that the four major phyla are *Actinobacteria*, *Firmicutes*, *Bacteroidetes*, and *Proteobacteria*^4,22–24^. Other studies also found among their major phyla *Fusobacteria* and *Tenericutes*^25^. The increase or high frequency of major phyla can be linked to the health of cattle. *Proteobacteria* and *Firmicutes* have the major proportion in healthy cows^26,27^. At the same time, the major proportion of *Fusobacteria* and *Bacteroidetes* were present in cow reproductive tracts that developed reproductive disease during postpartum^26–28^.

The most abundant phylum in UH and UB was *Bacteroidetes* which has a high abundance in cows with diseases in the reproductive tract. However, the studied cows were healthy, thus, it suggests that the cows are not affected by this highest proportion of *Bacteroidetes*. Peng, et al. ^26^ showed that healthy cows and cows with metritis have Bacteroidetes among their major phyla. Still, *Bacteroidetes*, *Peptostreptococcus*, and *Fusobacterium* were higher in cows with metritis compared to healthy cows^26^. The presence of these phyla alone does not indicate disease, but an increase in their abundance within an environment can be a potential risk factor for uterine diseases in cattle. Hence, tracking the abundance of *Bacteroidetes* in healthy animals could help to understand the beginning of reproduction problems such as metritis.

Samples came from Brown Swiss and Crossbreed which can add more variability to the study. However, Our results showed not significant difference between the breeds into the alpha diversity (Supplementary File 1, Table S10). Furthermore, A study in Holstein Friesian cattle obtained that the most frequent phyla were *Firmicutes, Tenericutes, Proteobacteria*, and *Bacteroidetes*. However, cows of different breeds also can contain similar microbiomes as Gyr cattle and Nellore beef cattle. Both of them showed a higher frequency of *Firmicutes, Bacteroidetes,* and *Proteobacteria*^22^.The same research group has shown that despite the fitness, geographical distance, and differences in animal handling, Gyr and Nellore present a similar phyla into their microbiome.

At the genus level, *Chryseobacterium* was the most abundant genus of both sites. This genus has a wide range of multiplication/survival sites such as food, water sources, animals and humans^29^ and presents several pathogenic species with a variety of virulence features^29^. However, no previous report on the microbiome composition of cattle reproductive tract mentioned *Chryseobacterium* as a predominant taxon. One reason for this could be the effect of the different diets, geographic locations, and even breeds^24^. Microorganisms from this genus can produce toxic compounds such as Lecithinase (Phospholipase C), which can damage the reproductive tract tissue, leading to haemolysis and membrane disruption^30^.

Another genus that has a high relative abundance of both sites is *Flavobacterium.* It is associated with women who carry an in vitro fertilization (IVF) pregnancy to a successful term^31^. Members of the genus Flavobacterium are distributed widely in nature and have been isolated from various habitats, and each year, this number steadily increases. The *Flavobacterium* genus currently has around 394 related species. In recent studies, *Flavobacterium* strains were isolated from raw chicken meat, raw goat meat, poultry soil, mastitis in cattle and vagina environments^32,33^. This genus was associated with a higher relative abundance in patients who achieved pregnancy outcomes than in patients who did not^34^. It was impossible to confirm the classification of the *Flavobacterium* species, and there are no studies of a specific function of species of this genus related to pregnancy success. Therefore, the isolation of the bacteria using an appropriate method may, in the future, classify this species in pregnant or non-pregnant cows. However, finding the genera *Chryseobacterium or Flavobacterium* does not define alone the total aspects of a positive pregnancy; we do know that several other aspects can influence that.

Additionally, our results showed some genera that were abundant in only one of the sites. One of them is an unclassified member of *Lachnospiraceae* (NK3A20 group) and its relative abundance was significantly higher in UB compared to UH. *Lachnospiraceae* was associated with a healthy vagina environment in bovines^35^. Previous studies report that this family is part of commensal bacteria in the bovine reproductive tract of dairy and beef cattle^36–38^.

Furthermore, *Oscillospiraceae* (NK4A214 group) was a genus significantly abundant in UB compared to UH based on the Heat tree analysis. *Oscillospiraceae* was suggested to have a beneficial effect on pregnancy outcome because of a mutualistic interaction with the host^39^. Therefore, this family has the potential to act as a biomarker for successful pregnancy. Another genus found with a higher abundance in UB than in UH was *Rhodococcus.* Some species inside this genus are important bacteria that cause severe infection and abortion in equines such as *Rhodococcus equi*^40^.

Also, the unique genus that was more abundant in UH than UB was *Paeniglutamicibacter* which is associated with cattle that do not have success to establish a pregnancy^39^. In our samples, it is shown that there is a high presence of exclusive genera in UB. One of the reasons for this result is that UB has more contact with the environment, especially during and after parturition^41^. Tissues with more contact with the external environment present a higher load of microorganisms such as skin, mouth, and gut^42^. This presence is often related to a higher diversity compared to tissues that are more isolated^25^. Another reason is the immune response and structure of the tissue. Comparison studies between the vagina and uterus showed that the vagina has a higher number of bacterial taxa even though they are part of the same system and are next to each other^23^. This is explained by the presence of the cervix acting like a barrier and the difference between their immune responses^23^. Biological barriers are shown to be important for controlling the microbiome of close structures, or keeping them isolated, for example, brain^43^ and eyes.

The distinctions between UB and UH have not been fully delineated^44^. However, a study conducted by Pothmann, et al. ^44^ demonstrated a significant mRNA expression of Interleukin 1 beta, Interleukin 1 alpha, and C-X-C motif chemokine ligand in UH. Additionally, UH creates an environment that is less favorable for bacterial growth and colonization due to the presence of antimicrobial substances^45^ and ciliary movement^46^, which aids in the elimination of potential pathogens.

Consequently, UH fosters a more controlled and restricted immune cell environment, potentially explaining the reduced diversity of exclusive taxa. Moreover, this finding suggests that even slight variations in the microbiota of UH can lead to significant disturbances in reproductive health. Furthermore, the identification of exclusive potential pathogen genera in UB, such as *Escherichia-Shigella*, *Fusobacterium*, and *Streptococcus*^47^, supports this hypothesis. Although the presence of these genera in UB does not appear to cause disturbances or affect the health of the cows, an increase in their proportions could pose a risk to reproductive health^48^. These findings show too, that the biological barrier works in favor of the internal environment, such as the UH.

This comparative analysis highlights how animal biology can be an obstacle to microbial colonization unless influenced by factors such as diseases, diet, and environment. Furthermore, this study contributes to our understanding of the microbiome in two distinct sites (UB and UH) of the reproductive tract in cattle from previously unexplored environments in the Amazon region (Peru). The beta diversity analysis reveals minimal variation between UH and UB, likely due to their proximity within the reproductive system. This is also evident in the shared genera observed in the Venn Diagram depicting the overlap between the sites. Moreover, our findings indicate that UB exhibits higher richness compared to UH, while UH demonstrates a lower number of unique taxa relative to UB. These observations suggest that the UH microbiota is more restricted, making even slight variations capable of inducing significant disturbances in the health of cows. Consequently, the UH microbiota holds promise as an excellent marker for identifying healthy cows, given its selectivity within the prevailing environment.

On the other hand, the microbiota in UB can retain many exclusive commensal bacteria, which can be shown in the high richness and diversity. In addition, some entire genera in UH are potential pathogen species related to endometriosis and other uterus diseases. However, all the cows showed a general and reproductive healthy phenotype. Hence, UB could be less affected by various pathogenic organisms. This demonstrates that through the evolution and adaptation of the cows, UH has become an environment of vast colonization due to the conditions offered by the breeder and reproductive period situations. This adaptation may enable these animals to be successfully farmed and domesticated with optimal reproductive success, even under adverse conditions.

There are studies in the female reproductive tract of cattle that showed variation by factors such as age, puberty, menstrual and estrous cycle^11^. In addition, samples were collected from different cows so we could have individual variation between samples and the correlation between UB and UH could be less clear. This is because microbiomes could have a wide difference between individuals even of the same species^22,36^. Hence, further studies can extract samples at different times of the year, or disease situations of the same cows to reduce the individual variation and consider the breed effect.

Further studies can use the knowledge of our results that can lead to other analyses related to reproductive features such as the success of embryonic implantation. Furthermore, these studies could consider variables such as estrous cycle, and puberty^11^. As we know, to achieve successful implantation, the uterus should undergo structural and functional remodeling^49^. Besides that, estrogen and progesterone are the master hormones mediating these changes, where these hormones bind to their respective nuclear receptors^49^. One of the multiple variables of the success of embryo implantation can be the microbiome^50^ and it will be important to study their composition. Overall, reproductive success is an important determinant of profitability for commercial cattle production. Hence, the study of the microbiome communities can generate a high impact on the industry, including in developing countries such as Peru.

In the present study, there were differences in the composition of taxa between the analyzed uterine sites. UH and UB microbiome diversity did not have significant differences. Both uterine sites have the same four most abundant phyla: *Actinobacteria*, *Bacteroidetes*, *Firmicutes*, and *Proteobacteria*. In addition, they share the same most abundant genus: *Chryseobacterium*. However, the other most abundant genera vary between uterine sites (UB and UH). UB presents a more significant number of unique taxa compared to UH. These results suggest that the composition of bacteria inside the uterus shows apparent differences, such as some exclusive and abundant genera. This demonstrates that well-established microbiota can prevent and control the excessive growth of a pathogenic microorganism to the point that it can have a high abundance. As well, biological barriers and functional parts can help to control the community of microorganisms. With this, we help understanding of biomarkers between uterine body and uterine horn of cows, prior reproduction period.

## Methods

### Ethics statement

The experimental protocol was approved by the Institutional Committee on Research Ethics of the UNTRM, according to protocol number CIEI-No. 012. All experiments were carried out in accordance with the approved guidelines and regulations. In addition, the methods were carried out in accordance with ARRIVE guidelines (Animal Research: Reporting of In Vivo Experiments).

### Criteria for selection, treatment, and sampling of animals

A complete gynecological evaluation of the reproductive system (vulva, vagina, cervix, UB, UHs and ovaries) was performed to determine if the animal was clinically healthy and with normal reproductive functions The cows were selected from the Olleros cattle basin in Amazon region, Peru, and analyzed in the Molecular Physiology Laboratory of the National University Toribio Rodríguez de Mendoza (UNTRM).

A total of 15 cows were used in this study, six of which were Brown Swiss and nine Crossbreed. The mean age of the cows was 3.5 ± 2.1 years and the body condition was greater or equal than 3 on a scale of 1 to 5 (1 meaning very skinny and 5 meaning very fat)^51^.

The samples were extracted with a stainless-steel gun with disposable cervical-uterine gynecological brushes and Cassou-type disposable sanitary sheaths for artificial insemination (AI) 20 days before starting the estrus synchronization protocol^52^. The cows were immobilized with cattle immobilizers in order to facilitate the extraction of the mucosa, besides cows with aggressive behavior were injected with 0,3–1 mg/kg Xylazine. After, the perineal and vulvar areas of each animal were cleaned and disinfected with 70% alcohol. A cytobrush attached to the AI gun was gently entered through the vulva of the cow, passing the cervix to the UB.

The cytobrush attached to the AI gun was inserted to the sample collection area (the UB and cranial half of the UH), the brush was exposed to the endometrial mucosal surface, and a scraping of the UB wall and the cranial half of the uterine horn was performed. The extracted mucosa was placed in a cryotube of 4 mL capacity with 2 mL of Phosphate-Buffer Saline (PBS). For microbiome analysis, samples were previously frozen in liquid nitrogen and stored at -80 °C in the Molecular Physiology Laboratory (UNTRM).

### DNA extraction, amplification, and sequencing of the 16S rRNA gene

DNA was extracted using the PureLink Genomic DNA Extraction MiniKit (Invitrogen, Life Technologies, CA, USA) following the recommended protocol of the manufacturer for Gram Positive Bacterial Cell Lysate with some modifications. To purify the extracted genomic DNA, the "DNA Clean and Concentrator^®^-5" kit (Zymo Research Corp., Irvine, CA, USA) was used. The concentration and purity of the DNA were quantified by spectroscopy (optical density) on a NanoDrop^®^ Spectrophotometer (Thermo Fisher Scientific, Wilmington, DE, USA) and verified with agarose gel electrophoresis.

In the Argonne Laboratory (Argonne, IL, USA), the V4 hypervariable region of the bacterial 16S rRNA gene was amplified from genomic DNA by Polymerase chain reaction using the primers 515 F and 806 R optimized for the Illumina MiSeq platform (Illumina Inc., San Diego, CA, USA)^53^ with MiSeq Reagent Kit V2 (Illumina Inc., San Diego, CA, USA). Degeneracy was added to both the forward and reverse primers to remove known biases against *Crenarchaeota*/*Thaumarchaeota* [515F, also called 515F-Y^54^] and the marine and freshwater Alphaproteobacterial clade SAR11 [806R^55^].

### Sequence and bioinformatics analyses

The samples were divided into two groups based on the uterine sites from which they were extracted: UB (samples from the uterine body) and UH (samples from the cranial half of the uterine horn). The microbiome analysis of the V4 hypervariable region of the 16S rRNA gene was done with the Quantitative Insights Into Microbial Ecology 2 (QIIME2) software (v. 2023.2)^56^. We followed the QIIME2 pipeline to perform the demultiplexing of the reads, the trimming process of the sequence adapters, and the elimination of ambiguous, duplicate, low-quality, chimera, and other sequences through the denoise-paired method using the ‘DADA2’ plugin (v. 1.26.0)^57^ to infer the amplicon sequence variant (ASV) present in each sample and continue with the analysis only up to positions 226 and 208 of the forward and reverse reads, respectively. In addition, alpha rarefaction was used to exclude sequences with insufficient ASVs per sample.

With the representative and high-quality sequences, the taxonomic classification was applied with the SILVA v. 138 database^58^ and the sklearn classifier, obtaining the taxonomy tables and ASVs. The package phyloseq^59^ in R^60^ was used to filter the data with the removal of any ASVs without a bacterial phylum assignment, assigned as *Archaea*, Chloroplast or Mitochondrial origin, or unassigned.

All statistical analyses were carried out by using several packages and functions implemented in R 4.2.2 ^56^. Alpha rarefaction curves were plotted with the package vegan^61^. To calculate bacterial diversity, the alpha diversity indices were analyzed in the phyloseq package^59^, using metrics of the indices Shannon diversity^62^, Chao1 richness^63^, Abundance-based Coverage Estimator (ACE) of species richness^64^ and Observed Species in the R statistical software. The alpha diversity box-and-whisker plots were designed with the same package. The index values for different collecting segments (UB and UH groups) were compared by Analysis of variance (ANOVA) (α < 0.05), followed by the Tukey's honestly-significant-difference (HSD) post hoc test using the package stats^60^.

Beta diversity analysis for dissimilarity in community structure between different collecting segments was assessed with principal coordinate ordination using weighted and unweighted UniFrac metrics, by performing non-metric multidimensional scaling (NMDS)^65^ and with canonical analysis of principal coordinates (CAP)^66^ in the packages phyloseq^59^ and vegan^61^. Deeper analysis with Permutational multivariate analysis of variance (PERMANOVA) were performed for differences in the communities among collecting segments, which were conducted using the function adonis2 from the package vegan^61^ and all the metrics mentioned above over 1000 permutations. Analysis of similarities (ANOSIM) and Multivariate homogeneity of group dispersions were also performed using the functions anosim and betadisper, respectively. Pairwise post hoc tests were conducted with the function pairwise.adonis from the package pairwiseAdonis^67^ with Euclidean method and Bonferroni correction to calculate the statistical significance.

The microbial composition in the stacked bar plots was analyzed using the packages qiime2R^68^ and ggplot2^69^ in R, in order to compare the taxonomic bar plots with relative and absolute abundance at phylum and genus levels. Linear discriminant analysis (LDA) effect size (LEfSe) analysis was performed through the package microeco^70^ to determine those taxa that demonstrated an LDA score > 2 and an LDA score < -2 for effect size within collecting segments, along with their relative abundances. The R package metacoder^71^ was used for representing the taxonomic abundance as a differential heat tree using a Wilcox rank-sum test followed by a Benjamin-Hochberg (FDR) correction for multiple comparisons. The packages MicrobiotaProcess^72^, zoo^73^ and VennDiagram^74^ were used to generate lists with the unique and shared taxa between the collecting segments, as well as generate a Venn Diagram with the different collecting segments.

### Supplementary Information

Supplementary Information 1.

Supplementary Information 2.

Supplementary Information 3.

Supplementary Information 4.

Supplementary Information 5.

## Data Availability

The DNA sequences generated and analyzed during the current study are available in the NCBI SRA repository under BioProject PRJNA974053 (https://www.ncbi.nlm.nih.gov/bioproject/PRJNA974053). Other data from the study are available from the corresponding author upon reasonable request.
