# Supplementary material for: Exploring the microbiome of two uterine sites in cows
Source: Sci Rep. 2023 Oct 31;13:18768. doi: 10.1038/s41598-023-46093-0 (PMC10618249; doi:10.1038/s41598-023-46093-0)
Supplement: Supplementary file 1 — Supplementary Information 1. [file 41598_2023_46093_MOESM1_ESM.docx]

**Supplementary File 1**

**Supplementary Table S1** – Metadata of the samples. It contains the ID number of the Cow (Cow_ID), SampleID, Barcode Sequence, Site (UH= Uterine Horn, UB= Uterine Body), N° parturition (Number of births that cows had during their lives.), District (O = Olleros) and Breed (BS = Brow Swiss, Cb = Crossbreed).

| **Cow_ID** | **SampleID** | **Barcode_Sequence** | **Site** | **N° parturition** | **Breed** | **District** |
| --- | --- | --- | --- | --- | --- | --- |
| 10 | C10_S19 | GTTATGACGGAT | UH | 4 | BS | O |
| 12 | C12_S20 | TAGTACTATACT | UH | 1 | BS | O |
| 15 | C15_S21 | GTGAACTGGATT | UH | 7 | Cb | O |
| 17 | C17_S22 | ATAAGGTCGCCT | UH | 6 | Cb | O |
| 18 | C18_S23 | CGTTAGTGACTG | UH | 3 | Cb | O |
| 43 | C43_S28 | TCGCATGGATAC | UH | 1 | Cb | O |
| 45 | C45_S29 | AGTTTAATCTCA | UH | 1 | Cb | O |
| 10 | U10_S4 | TTGTATGACAGG | UB | 4 | BS | O |
| 12 | U12_S6 | ACGAGGAGTCGA | UB | 1 | BS | O |
| 13 | U13_S5 | AACCCTAACTGG | UB | 4 | Cb | O |
| 14 | U14_S7 | CGATCACCACAA | UB | 7 | BS | O |
| 15 | U15_S8 | TGTACGGATAAC | UB | 7 | Cb | O |
| 18 | U18_S9 | GGTTCCATTAGG | UB | 3 | Cb | O |
| 5 | U5_S2 | GATGTCATAGCC | UB | 2 | Cb | O |
| 7 | U7_S3 | GAGGGCGTGATC | UB | 1 | BS | O |

**Supplementary Table S2** – Alpha Diversity Analysis – Significance test of ANOVA for Shannon and Chao1 indices for Site groups.

| **ANOVA** | | | | | | |
| --- | --- | --- | --- | --- | --- | --- |
| **Summary (aov. Shannon)** | Df | Sum-Sq | Mean-Sq | F-value | Pr(>F) | sig |
| **Site** | 1 | 0.115 | 0.1154 | 0.245 | 0.629 | ‘’ |
| **Residuals** | 13 | 6.114 | 0.4703 |  |  |  |
| **Summary (aov. Chao1)** | Df | Sum-Sq | Mean-Sq | F-value | Pr(>F) |  |
| **Site** | 1 | 3253 | 3253 | 3.08 | 0.103 | ‘’ |
| **Residuals** | 13 | 13731 | 1056 |  |  |  |

Signif. codes: |0 = ‘***’| - |0.001 = ‘**’| - |0.01 = ‘*’| - |0.05 = ‘.’| - |0.1 = ‘’ | - |1|

**Supplementary Table S3** **-** Alpha Diversity Analysis for index Shannon- Tukey’s honest significance test of our ANOVA - Tukey multiple comparisons of means - 95% family-wise confidence level for Site groups.

| **Shannon** | | | | | |
| --- | --- | --- | --- | --- | --- |
| **Site** | Diff | Lwr | Upr | p | Sig |
| **UB vs UH** | -0.1757847 | -0.9425712 | 0.5910019 | 0.6286814 | ‘’ |

Signif. codes: |0 = ‘***’| - |0.001 = ‘**’| - |0.01 = ‘*’| - |0.05 = ‘.’| - |0.1 = ‘’ | - |1|

**Supplementary Table S4** **-** Analysis of Alpha Diversity for index Chao1- Tukey's honest significance test of our ANOVA - Tukey multiple comparisons of means - 95% family-wise confidence level for Site groups

| **Chao1** | | | | | |
| --- | --- | --- | --- | --- | --- |
| **Site** | Diff | Lwr | Upr | p | sig |
| **UB vs UH** | -29.51786 | -65.85532 | 6.81961 | 0.1027947 | ‘’ |

Signif. codes: |0 = ‘***’| - |0.001 = ‘**’| - |0.01 = ‘*’| - |0.05 = ‘.’| - |0.1 = ‘’ | - |1|

**Supplementary Table S5** - Statistical analysis of beta diversity. Pairwise Permanova, considering the Unweighted Unifrac method in the calculation, and with the Bonferroni correction for comparing Site groups.

| **Beta Diversity** | | | | | | | | |
| --- | --- | --- | --- | --- | --- | --- | --- | --- |
| Site | Df | Sums.Of.Sqs | F.Model | R2 | p-value | sig | p.adjusted | sig |
| **UB vs UH** | 1 | 0.06020068 | 0.5489131 | 0.04051344 | 0.666 | ‘’ | 0.666 | ‘’ |

Signif. codes: |0 = ‘***’| - |0.001 = ‘**’| - |0.01 = ‘*’| - |0.05 = ‘.’| - |0.1 = ‘’ | - |1|

**Supplementary Table S6** – Alpha Diversity Analysis – Significance test of ANOVA for Shannon and Chao1 indices using like independent variable the cow that sample were extracted.

| **ANOVA** | | | | | | |
| --- | --- | --- | --- | --- | --- | --- |
| **Summary (aov. Shannon)** | Df | Sum-Sq | Mean-Sq | F-value | Pr(>F) | sig |
| **Cow_ID** | 10 | 5.111 | 0.5111 | 1.827 | 0.295 | ‘’ |
| **Residuals** | 4 | 1.119 | 0.2797 |  |  |  |
| **Summary (aov. Chao1)** | Df | Sum-Sq | Mean-Sq | F-value | Pr(>F) |  |
| **Cow_ID** | 10 | 12313 | 1231 | 1.054 | 0.524 | ‘’ |
| **Residuals** | 4 | 4671 | 1168 |  |  |  |

Signif. codes: |0 = ‘***’| - |0.001 = ‘**’| - |0.01 = ‘*’| - |0.05 = ‘.’| - |0.1 = ‘’ | - |1|

**Supplementary Table S7** **-** Alpha Diversity Analysis for index Shannon- Tukey’s honest significance test of our ANOVA - Tukey multiple comparisons of means - 95% family-wise confidence level for cow groups.

| **Shannon** | | | | | |
| --- | --- | --- | --- | --- | --- |
| **Cow** | **diff** | **lwr** | **upr** | **p adj** | **Sig** |
| 12-10 | 1.4423495 | -1.559561 | 4.44426 | 0.3969885 | " |
| 13-10 | -0.14276948 | -3.819343 | 3.533805 | 0.9999999 | " |
| 14-10 | -0.3874811 | -4.064055 | 3.289093 | 0.9995214 | " |
| 15-10 | 0.44035937 | -2.561551 | 3.442269 | 0.9943668 | " |
| 17-10 | 0.92713613 | -2.749438 | 4.60371 | 0.8890657 | " |
| 18-10 | 0.46082515 | -2.541085 | 3.462735 | 0.9923618 | " |
| 43-10 | 0.75412051 | -2.922453 | 4.430695 | 0.9576229 | " |
| 45-10 | -0.28968646 | -3.96626 | 3.386888 | 0.999959 | " |
| 05-10 | 1.01504545 | -2.661529 | 4.691619 | 0.8421493 | " |
| 07-10 | 1.20172526 | -2.474849 | 4.878299 | 0.7267503 | " |
| 13-12 | -1.58511898 | -5.261693 | 2.091455 | 0.4894269 | " |
| 14-12 | -1.8298306 | -5.506405 | 1.846743 | 0.3684975 | " |
| 15-12 | -1.00199013 | -4.0039 | 1.99992 | 0.7102285 | " |
| 17-12 | -0.51521337 | -4.191787 | 3.161361 | 0.9958917 | " |
| 18-12 | -0.98152435 | -3.983434 | 2.020386 | 0.7264967 | " |
| 43-12 | -0.68822899 | -4.364803 | 2.988345 | 0.974195 | " |
| 45-12 | -1.73203596 | -5.40861 | 1.944538 | 0.4133214 | " |
| 05-12 | -0.42730405 | -4.103878 | 3.24927 | 0.998965 | " |
| 07-12 | -0.24062424 | -3.917198 | 3.43595 | 0.9999922 | " |
| 14-13 | -0.24471162 | -4.490054 | 4.00063 | 0.9999976 | " |
| 15-13 | 0.58312885 | -3.093445 | 4.259703 | 0.990547 | " |
| 17-13 | 1.06990561 | -3.175436 | 5.315248 | 0.8893472 | " |
| 18-13 | 0.60359463 | -3.072979 | 4.280169 | 0.9882227 | " |
| 43-13 | 0.89688999 | -3.348452 | 5.142232 | 0.9507082 | " |
| 45-13 | -0.14691698 | -4.392259 | 4.098425 | 1 | " |
| 01-05 | 1.15781493 | -3.087527 | 5.403157 | 0.8491289 | " |
| 01-07 | 1.34449474 | -2.900847 | 5.589837 | 0.7508348 | " |
| 15-14 | 0.82784047 | -2.848734 | 4.504414 | 0.9327441 | " |
| 17-14 | 1.31461723 | -2.930725 | 5.559959 | 0.7673323 | " |
| 18-14 | 0.84830625 | -2.828268 | 4.52488 | 0.9246601 | " |
| 43-14 | 1.14160161 | -3.10374 | 5.386944 | 0.8569228 | " |
| 45-14 | 0.09779464 | -4.147547 | 4.343137 | 1 | " |
| 01-05 | 1.40252655 | -2.842815 | 5.647869 | 0.7183865 | " |
| 01-07 | 1.58920636 | -2.656136 | 5.834548 | 0.6139952 | " |
| 17-15 | 0.48677676 | -3.189797 | 4.163351 | 0.997257 | " |
| 18-15 | 0.02046578 | -2.981444 | 3.022376 | 1 | " |
| 43-15 | 0.31376115 | -3.362813 | 3.990335 | 0.9999177 | " |
| 45-15 | -0.73004583 | -4.40662 | 2.946528 | 0.9642896 | " |
| 01-05 | 0.57468608 | -3.101888 | 4.25126 | 0.9914003 | " |
| 01-07 | 0.76136589 | -2.915208 | 4.43794 | 0.9554761 | " |
| 18-17 | -0.46631098 | -4.142885 | 3.210263 | 0.9979966 | " |
| 43-17 | -0.17301562 | -4.418358 | 4.072326 | 0.9999999 | " |
| 45-17 | -1.21682259 | -5.462165 | 3.028519 | 0.8195549 | " |
| 01-05 | 0.08790932 | -4.157433 | 4.333251 | 1 | " |
| 01-07 | 0.27458913 | -3.970753 | 4.519931 | 0.999993 | " |
| 43-18 | 0.29329536 | -3.383279 | 3.969869 | 0.9999542 | " |
| 45-18 | -0.75051161 | -4.427086 | 2.926062 | 0.9586679 | " |
| 01-05 | 0.5542203 | -3.122354 | 4.230794 | 0.9932311 | " |
| 01-07 | 0.74090011 | -2.935674 | 4.417474 | 0.9613723 | " |
| 45-43 | -1.04380697 | -5.289149 | 3.201535 | 0.9002289 | " |
| 01-05 | 0.26092493 | -3.984417 | 4.506267 | 0.9999956 | " |
| 01-07 | 0.44760475 | -3.797737 | 4.692947 | 0.9995198 | " |
| 01-05 | 1.30473191 | -2.94061 | 5.550074 | 0.7727469 | " |
| 01-07 | 1.49141172 | -2.75393 | 5.736754 | 0.6683515 | " |
| 07-05 | 0.18667982 | -4.058662 | 4.432022 | 0.9999998 | " |

Signif. codes: |0 = ‘***’| - |0.001 = ‘**’| - |0.01 = ‘*’| - |0.05 = ‘.’| - |0.1 = ‘’ | - |1|

**Supplementary Table S8** **-** Analysis of Alpha Diversity for index Chao1- Tukey's honest significance test of our ANOVA - Tukey multiple comparisons of means - 95% family-wise confidence level for cow groups

| **Chao1** | | | | | |
| --- | --- | --- | --- | --- | --- |
| **Cow** | **diff** | **lwr** | **upr** | **p adj** | **Sig** |
| 12-10 | 4.50E+00 | -189.463 | 198.463 | 1 | " |
| 13-10 | -8.00E+00 | -245.5551 | 229.5551 | 1 | " |
| 14-10 | -1.70E+01 | -254.5551 | 220.5551 | 0.9999826 | " |
| 15-10 | 3.90E+01 | -154.963 | 232.963 | 0.9618262 | " |
| 17-10 | -1.50E+01 | -252.5551 | 222.5551 | 0.9999944 | " |
| 18-10 | -8.50E+00 | -202.463 | 185.463 | 0.9999998 | " |
| 43-10 | -9.00E+00 | -246.5551 | 228.5551 | 1 | " |
| 45-10 | -1.70E+01 | -254.5551 | 220.5551 | 0.9999826 | " |
| 05-10 | -1.20E+01 | -249.5551 | 225.5551 | 0.9999993 | " |
| 07-10 | 9.20E+01 | -145.5551 | 329.5551 | 0.5842137 | " |
| 13-12 | -1.25E+01 | -250.0551 | 225.0551 | 0.999999 | " |
| 14-12 | -2.15E+01 | -259.0551 | 216.0551 | 0.9998641 | " |
| 15-12 | 3.45E+01 | -159.463 | 228.463 | 0.9807961 | " |
| 17-12 | -1.95E+01 | -257.0551 | 218.0551 | 0.9999413 | " |
| 18-12 | -1.30E+01 | -206.963 | 180.963 | 0.9999904 | " |
| 43-12 | -1.35E+01 | -251.0551 | 224.0551 | 0.9999979 | " |
| 45-12 | -2.15E+01 | -259.0551 | 216.0551 | 0.9998641 | " |
| 05-12 | -1.65E+01 | -254.0551 | 221.0551 | 0.9999867 | " |
| 07-12 | 8.75E+01 | -150.0551 | 325.0551 | 0.6280298 | " |
| 14-13 | -9.00E+00 | -283.3051 | 265.3051 | 1 | " |
| 15-13 | 4.70E+01 | -190.5551 | 284.5551 | 0.9649774 | " |
| 17-13 | -7.00E+00 | -281.3051 | 267.3051 | 1 | " |
| 18-13 | -5.00E-01 | -238.0551 | 237.0551 | 1 | " |
| 43-13 | -1.00E+00 | -275.3051 | 273.3051 | 1 | " |
| 45-13 | -9.00E+00 | -283.3051 | 265.3051 | 1 | " |
| 01-05 | -4.00E+00 | -278.3051 | 270.3051 | 1 | " |
| 01-07 | 1.00E+02 | -174.3051 | 374.3051 | 0.6369153 | " |
| 15-14 | 5.60E+01 | -181.5551 | 293.5551 | 0.9169751 | " |
| 17-14 | 2.00E+00 | -272.3051 | 276.3051 | 1 | " |
| 18-14 | 8.50E+00 | -229.0551 | 246.0551 | 1 | " |
| 43-14 | 8.00E+00 | -266.3051 | 282.3051 | 1 | " |
| 45-14 | 7.11E-15 | -274.3051 | 274.3051 | 1 | " |
| 01-05 | 5.00E+00 | -269.3051 | 279.3051 | 1 | " |
| 01-07 | 1.09E+02 | -165.3051 | 383.3051 | 0.5614955 | " |
| 17-15 | -5.40E+01 | -291.5551 | 183.5551 | 0.9296819 | " |
| 18-15 | -4.75E+01 | -241.463 | 146.463 | 0.9019126 | " |
| 43-15 | -4.80E+01 | -285.5551 | 189.5551 | 0.9608239 | " |
| 45-15 | -5.60E+01 | -293.5551 | 181.5551 | 0.9169751 | " |
| 01-05 | -5.10E+01 | -288.5551 | 186.5551 | 0.9465846 | " |
| 01-07 | 5.30E+01 | -184.5551 | 290.5551 | 0.9356084 | " |
| 18-17 | 6.50E+00 | -231.0551 | 244.0551 | 1 | " |
| 43-17 | 6.00E+00 | -268.3051 | 280.3051 | 1 | " |
| 45-17 | -2.00E+00 | -276.3051 | 272.3051 | 1 | " |
| 01-05 | 3.00E+00 | -271.3051 | 277.3051 | 1 | " |
| 01-07 | 1.07E+02 | -167.3051 | 381.3051 | 0.5778651 | " |
| 43-18 | -5.00E-01 | -238.0551 | 237.0551 | 1 | " |
| 45-18 | -8.50E+00 | -246.0551 | 229.0551 | 1 | " |
| 01-05 | -3.50E+00 | -241.0551 | 234.0551 | 1 | " |
| 01-07 | 1.01E+02 | -137.0551 | 338.0551 | 0.5060627 | " |
| 45-43 | -8.00E+00 | -282.3051 | 266.3051 | 1 | " |
| 01-05 | -3.00E+00 | -277.3051 | 271.3051 | 1 | " |
| 01-07 | 1.01E+02 | -173.3051 | 375.3051 | 0.6283403 | " |
| 01-05 | 5.00E+00 | -269.3051 | 279.3051 | 1 | " |
| 01-07 | 1.09E+02 | -165.3051 | 383.3051 | 0.5614955 | " |
| 07-05 | 1.04E+02 | -170.3051 | 378.3051 | 0.6028713 | " |

Signif. codes: |0 = ‘***’| - |0.001 = ‘**’| - |0.01 = ‘*’| - |0.05 = ‘.’| - |0.1 = ‘’ | - |1|

**Supplementary Table S9** - Statistical analysis of beta diversity. Pairwise Adonis, considering the Unweighted Unifrac method in the calculation.

| **Pairs** | **Df** | **SumsOfSqs** | **F.Model** | **R2** | **p.value** | **p.adjusted** | **sig** |
| --- | --- | --- | --- | --- | --- | --- | --- |
| 10 vs 12 | 1 | 0.4504186 | 2.4826515 | 0.5538355 | 0.3333333 | 1 |  |
| 10 vs 15 | 1 | 0.3481032 | 1.1968047 | 0.3743753 | 0.3333333 | 1 |  |
| 10 vs 17 | 1 | 0.3854842 | 1.5878729 | 0.6135823 | 0.3333333 | 1 |  |
| 10 vs 18 | 1 | 0.426473 | 1.9101203 | 0.4885068 | 0.3333333 | 1 |  |
| 10 vs 43 | 1 | 0.3865628 | 1.5923161 | 0.6142446 | 0.3333333 | 1 |  |
| 10 vs 45 | 1 | 0.569925 | 2.3476153 | 0.7012799 | 0.3333333 | 1 |  |
| 10 vs 13 | 1 | 0.3266523 | 1.3455349 | 0.573658 | 0.3333333 | 1 |  |
| 10 vs 14 | 1 | 0.3308125 | 1.3626712 | 0.5767503 | 0.6666667 | 1 |  |
| 10 vs 5 | 1 | 0.2871183 | 1.1826879 | 0.5418493 | 0.6666667 | 1 |  |
| 10 vs 7 | 1 | 0.4140755 | 1.7056454 | 0.6304024 | 0.3333333 | 1 |  |
| 12 vs 15 | 1 | 0.5476393 | 2.386028 | 0.5440066 | 0.3333333 | 1 |  |
| 12 vs 17 | 1 | 0.3111435 | 2.5910233 | 0.7215278 | 0.3333333 | 1 |  |
| 12 vs 18 | 1 | 0.3397044 | 2.0978596 | 0.5119403 | 0.3333333 | 1 |  |
| 12 vs 43 | 1 | 0.3083524 | 2.56778 | 0.7197137 | 0.3333333 | 1 |  |
| 12 vs 45 | 1 | 0.503322 | 4.1913744 | 0.8073728 | 0.3333333 | 1 |  |
| 12 vs 13 | 1 | 0.3099406 | 2.581006 | 0.7207489 | 0.3333333 | 1 |  |
| 12 vs 14 | 1 | 0.3391004 | 2.8238322 | 0.7384823 | 0.3333333 | 1 |  |
| 12 vs 5 | 1 | 0.1773871 | 1.477177 | 0.5963147 | 0.6666667 | 1 |  |
| 12 vs 7 | 1 | 0.498169 | 4.1484635 | 0.8057673 | 0.3333333 | 1 |  |
| 15 vs 17 | 1 | 0.4134102 | 1.2196671 | 0.549482 | 0.3333333 | 1 |  |
| 15 vs 18 | 1 | 0.5186117 | 1.911136 | 0.4886396 | 0.3333333 | 1 |  |
| 15 vs 43 | 1 | 0.4307878 | 1.2709355 | 0.5596528 | 0.3333333 | 1 |  |
| 15 vs 45 | 1 | 0.5339853 | 1.5753949 | 0.61171 | 0.3333333 | 1 |  |
| 15 vs 13 | 1 | 0.3700423 | 1.0917207 | 0.5219247 | 0.6666667 | 1 |  |
| 15 vs 14 | 1 | 0.4144077 | 1.2226102 | 0.5500785 | 0.3333333 | 1 |  |
| 15 vs 5 | 1 | 0.3871759 | 1.1422693 | 0.5332053 | 0.6666667 | 1 |  |
| 15 vs 7 | 1 | 0.302193 | 0.8915476 | 0.4713324 | 0.6666667 | 1 |  |
| 17 vs 18 | 1 | 0.2996428 | 1.4704747 | 0.5952195 | 0.3333333 | 1 |  |
| 17 vs 43 | 1 | 0.2811676 | NA | 1 | NA | NA |  |
| 17 vs 45 | 1 | 0.4627649 | NA | 1 | NA | NA |  |
| 17 vs 13 | 1 | 0.2593214 | NA | 1 | NA | NA |  |
| 17 vs 14 | 1 | 0.158853 | NA | 1 | NA | NA |  |
| 17 vs 5 | 1 | 0.2830069 | NA | 1 | NA | NA |  |
| 17 vs 7 | 1 | 0.4467126 | NA | 1 | NA | NA |  |
| 18 vs 43 | 1 | 0.3982291 | 1.9542793 | 0.661508 | 0.3333333 | 1 |  |
| 18 vs 45 | 1 | 0.5504098 | 2.7010949 | 0.7298097 | 0.3333333 | 1 |  |
| 18 vs 13 | 1 | 0.3953428 | 1.9401149 | 0.6598772 | 0.3333333 | 1 |  |
| 18 vs 14 | 1 | 0.1870019 | 0.9176976 | 0.4785413 | 0.6666667 | 1 |  |
| 18 vs 5 | 1 | 0.3247015 | 1.5934483 | 0.614413 | 0.3333333 | 1 |  |
| 18 vs 7 | 1 | 0.484509 | 2.3776915 | 0.7039398 | 0.3333333 | 1 |  |
| 43 vs 45 | 1 | 0.3846498 | NA | 1 | NA | NA |  |
| 43 vs 13 | 1 | 0.2534734 | NA | 1 | NA | NA |  |
| 43 vs 14 | 1 | 0.3020692 | NA | 1 | NA | NA |  |
| 43 vs 5 | 1 | 0.1902331 | NA | 1 | NA | NA |  |
| 43 vs 7 | 1 | 0.3907452 | NA | 1 | NA | NA |  |
| 45 vs 13 | 1 | 0.4892448 | NA | 1 | NA | NA |  |
| 45 vs 14 | 1 | 0.4156231 | NA | 1 | NA | NA |  |
| 45 vs 5 | 1 | 0.3789491 | NA | 1 | NA | NA |  |
| 45 vs 7 | 1 | 0.4589219 | NA | 1 | NA | NA |  |
| 13 vs 14 | 1 | 0.3060772 | NA | 1 | NA | NA |  |
| 13 vs 5 | 1 | 0.2612807 | NA | 1 | NA | NA |  |
| 13 vs 7 | 1 | 0.4227068 | NA | 1 | NA | NA |  |
| 14 vs 5 | 1 | 0.3047483 | NA | 1 | NA | NA |  |
| 14 vs 7 | 1 | 0.4410489 | NA | 1 | NA | NA |  |
| 5 vs 7 | 1 | 0.4059974 | NA | 1 | NA | NA |  |

Signif. codes: |0 = ‘***’| - |0.001 = ‘**’| - |0.01 = ‘*’| - |0.05 = ‘.’| - |0.1 = ‘’ | - |1|

**Supplementary Table S10** – Alpha Diversity Analysis – T-test results of differences of Shannon and Chao1 indices grouped by breed.

| **T-Student (two.sided)** | | | | |
| --- | --- | --- | --- | --- |
| **Shannon** | Df | t | Pr(>F) | sig |
| **Breed** | 13 | 0.45498 | 0.6566 | ‘’ |
| **Chao1** | Df | t | Pr(>F) | sig |
| **Breed** | 13 | 0.75067 | 0.4662 | ‘’ |

Signif. codes: |0 = ‘***’| - |0.001 = ‘**’| - |0.01 = ‘*’| - |0.05 = ‘.’| - |0.1 = ‘’ | - |1|

**Supplementary figure S1**


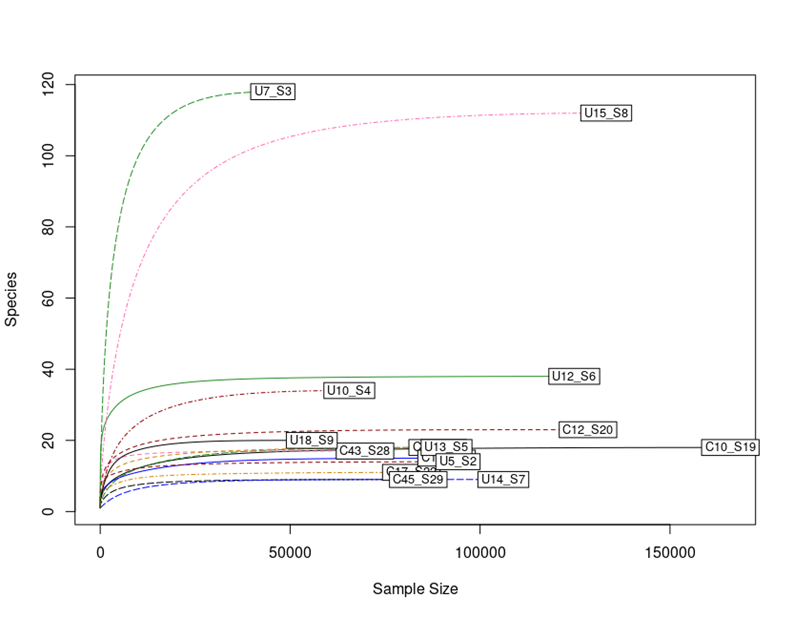


Alpha rarefaction curves. The rarefaction curves of each sample were indicated with different colors and types of lines. The letter C in the name of the samples stands for the samples from the UH group, and the letter U for the samples of the UB group.

**Supplementary figure S2**


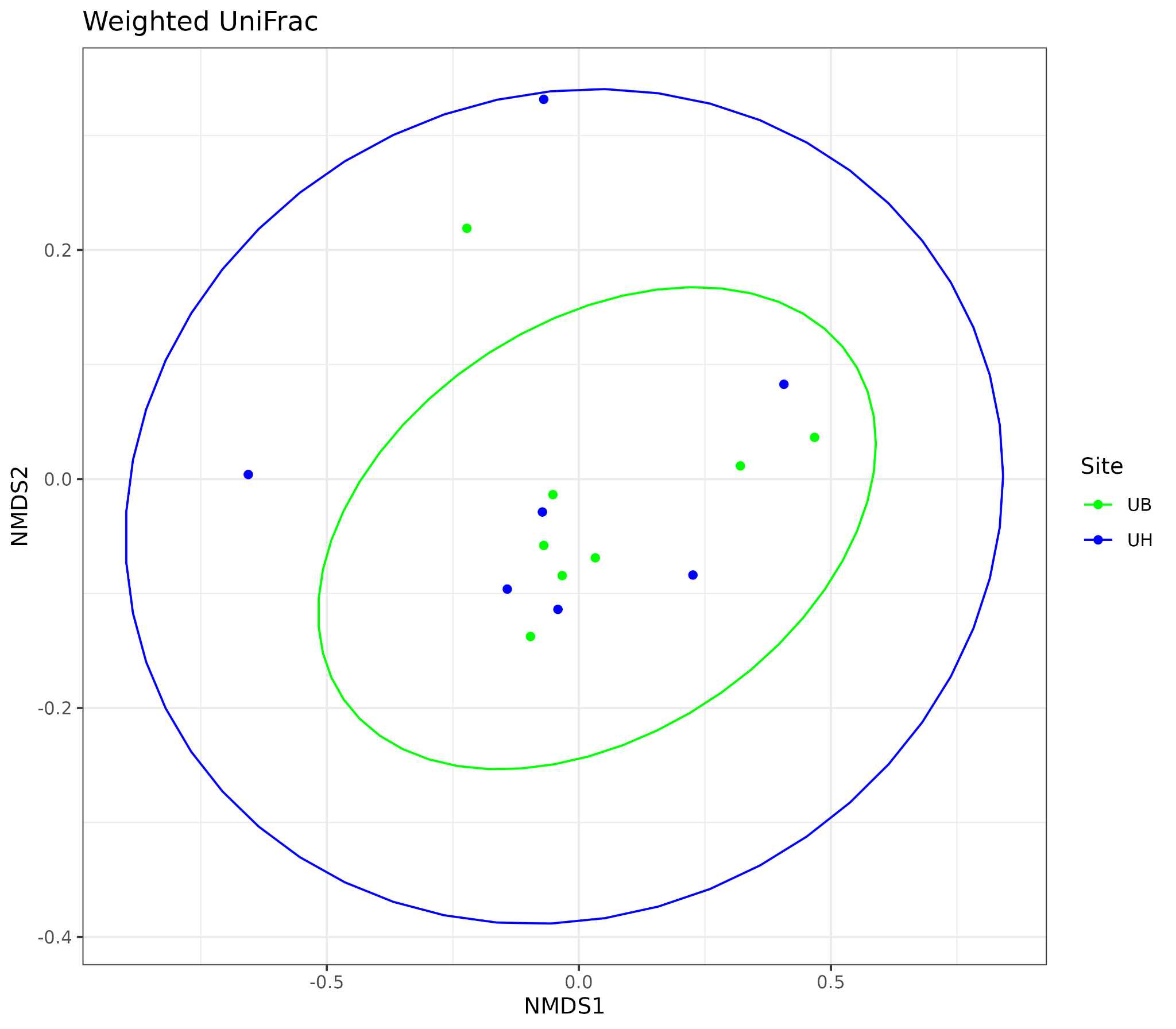


Unifrac weighted distance-based NMDS plot explains the phylogenetic relationship between species for beta diversity concerning site groups. Each color depicts a different group or type of site. The UniFrac weighted distance uses species abundance information and weights the branch length with the difference in abundance.

**Supplementary figure S3**


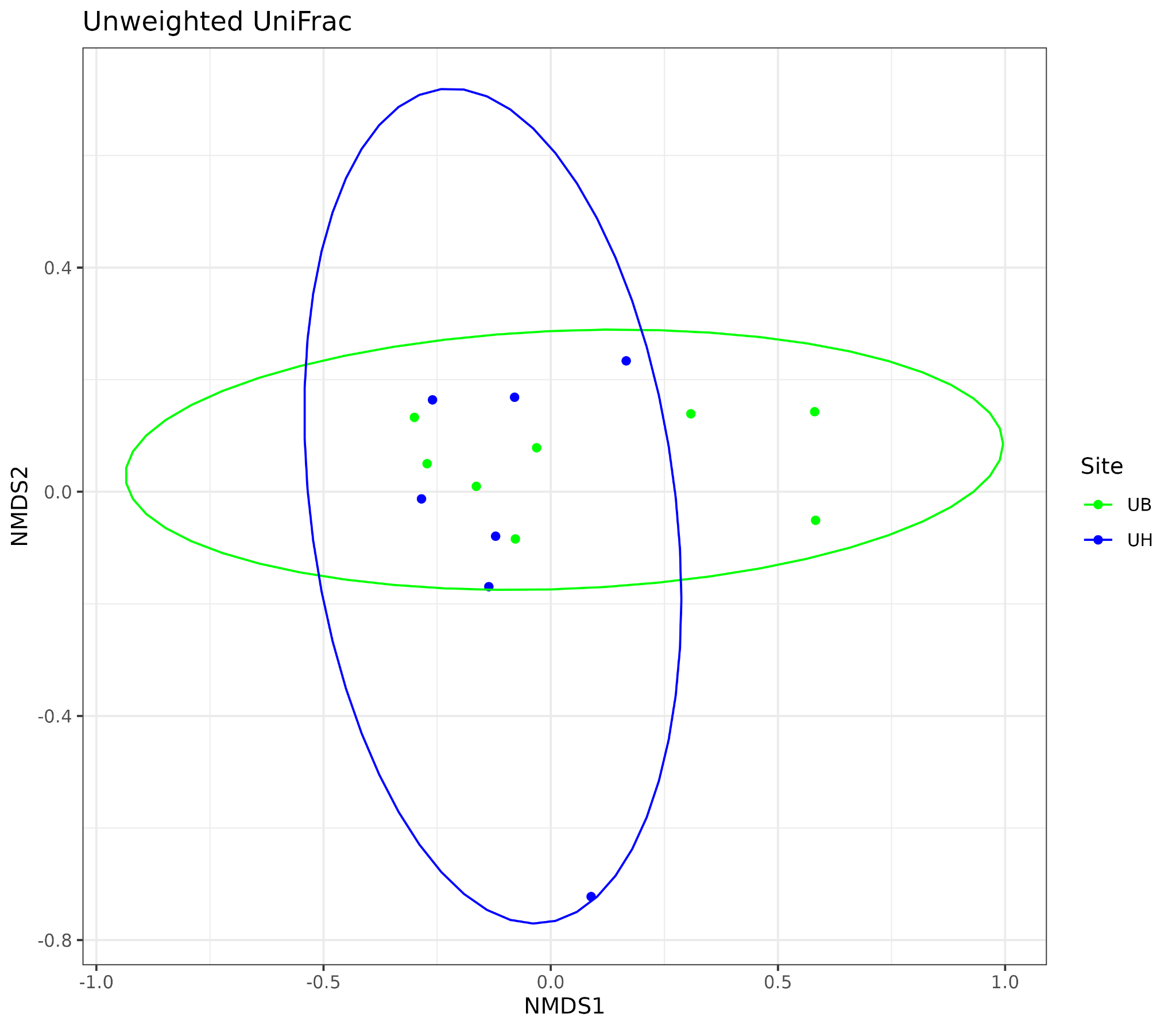


Unifrac unweighted distance-based NMDS plot explains the phylogenetic relationship between species for beta diversity concerning site groups. Each color depicts a different group. The UniFrac unweighted distance considers only the presence and absence of species information and counts the fraction of the length of the single branch for each community.

**Supplementary figure S4**


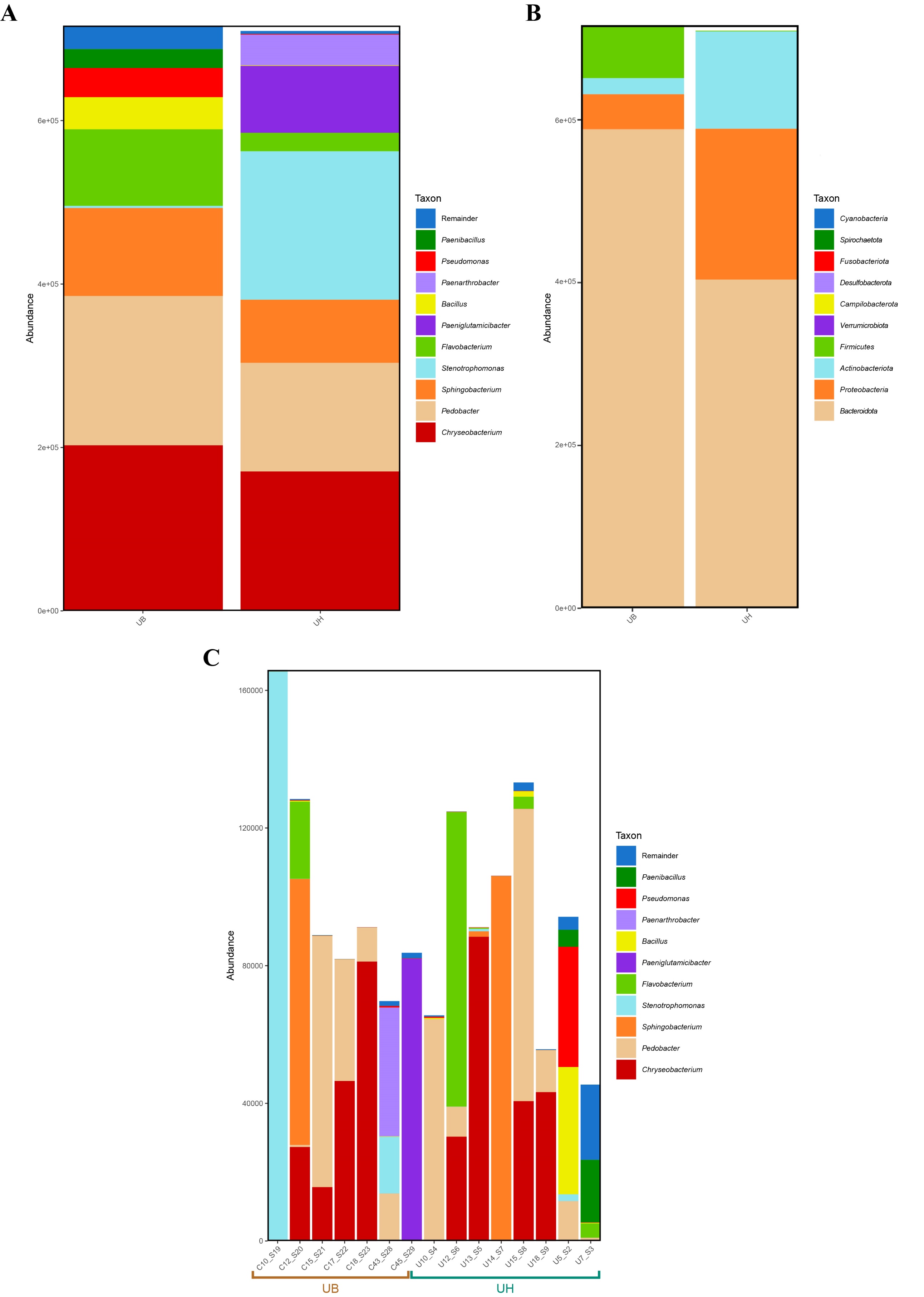


Bacterial microbiota composition in terms of absolute abundance at phylum and genus levels in the groups of sites (UB and UH): (A) Taxonomic composition of the ten main bacterial genera with different abundances among the different types of sites, with each color corresponding to a different genus. (B) Taxonomic composition of the ten main phyla and differentially abundant bacterial taxa, with each color corresponding to a phylum, in the different types of sites. (C) Taxonomic composition of the ten main genera and differentially abundant bacterial taxa, with each color corresponding to a different genus and subdivided by independent samples related to site (UB and UH).
